# Supplementary material for: Brief exposure of neuronal cells to levels of SCFAs observed in human systemic circulation impair lipid metabolism resulting in apoptosis
Source: Sci Rep. 2022 Aug 23;12:14355. doi: 10.1038/s41598-022-18363-w (PMC9399085; doi:10.1038/s41598-022-18363-w)
Supplement: Supplementary file 1 — Supplementary Figures. [file 41598_2022_18363_MOESM1_ESM.pdf]

Brief exposure of neuronal cells to levels of SCFAs observed in human systemic circulation impair lipid metabolism resulting in apoptosis

Tiffany A. Fillier*[1], Shrushti Shah[2], Karen M. Doody[3], Thu H. Pham[4], Isabelle Aubry[5], Michel L. Tremblay[5], Sukhinder K. Cheema[6], Jacqueline Blundell[7], Raymond H. Thomas[1,4]

[1]Department of Environmental Science, Memorial University of Newfoundland, St. John's, NL, Canada; [2]Department of Kinesiology, University of Calgary, Calgary, Alberta, Canada; [3]General Science Program and Aging Research Centre -Newfoundland and Labrador, Grenfell Campus, Memorial University of Newfoundland, Corner Brook, NL, Canada; [4]Boreal Ecosystem Research Initiative, Grenfell Campus, Memorial University of Newfoundland, Corner Brook, NL, Canada; [5]Rosalind and Morris Goodman Cancer Centre, McGill University, Montreal, QC, Canada; [6]Department of Biochemistry, Memorial University of Newfoundland, St. John's NL, Canada; [7]Department of Psychology, Memorial University of Newfoundlan d, St. John's , NL, Canada

[1*]tfillier@grenfell.mun.ca, rthomas@grenfell.mun.ca, [2]shrushti.shah@ucalgary.ca, [3]kdoody@grenfell.mun.ca, [4]tpham@grenfell.mun.ca, [5]isabelle.aubry@mcgill.ca, [5]michel.tremblay@mcgill.ca, [6]skaur@mun.ca, [7]jblundell@mun.ca

A)

Features	p-value	Significant	MC	MT	FC	FT
CL(20:4/18:1/18:1/22:6)	0.018	Yes	3.728 (a)	3.618 (a)	3.567 (a)	4.050 (b)
**CL(20:4/18:1/20:4/18:2)	0.018	Yes	3.626 (b)	3.893 (b)	3.157 (a)	3.803 (b)
CL(22:6/18:1/18:1/18:1)	0.040	Yes	4.554 (a)	4.610 (a)	4.681 (a)	4.946 (b)
**CL(22:1/20:4/20:4/18:0)	0.040	Yes	4.003 (b)	3.811 (ab)	4.176 (b)	3.518 (a)
CL(23:0/16:0/18:0/22:6)	0.040	Yes	4.862 (b)	5.031 (b)	5.220 (b)	4.331 (a)
CL(20:4/18:1/18:1/20:4)	0.043	Yes	5.414 (a)	5.704 (a)	5.632 (a)	6.123 (b)

B)

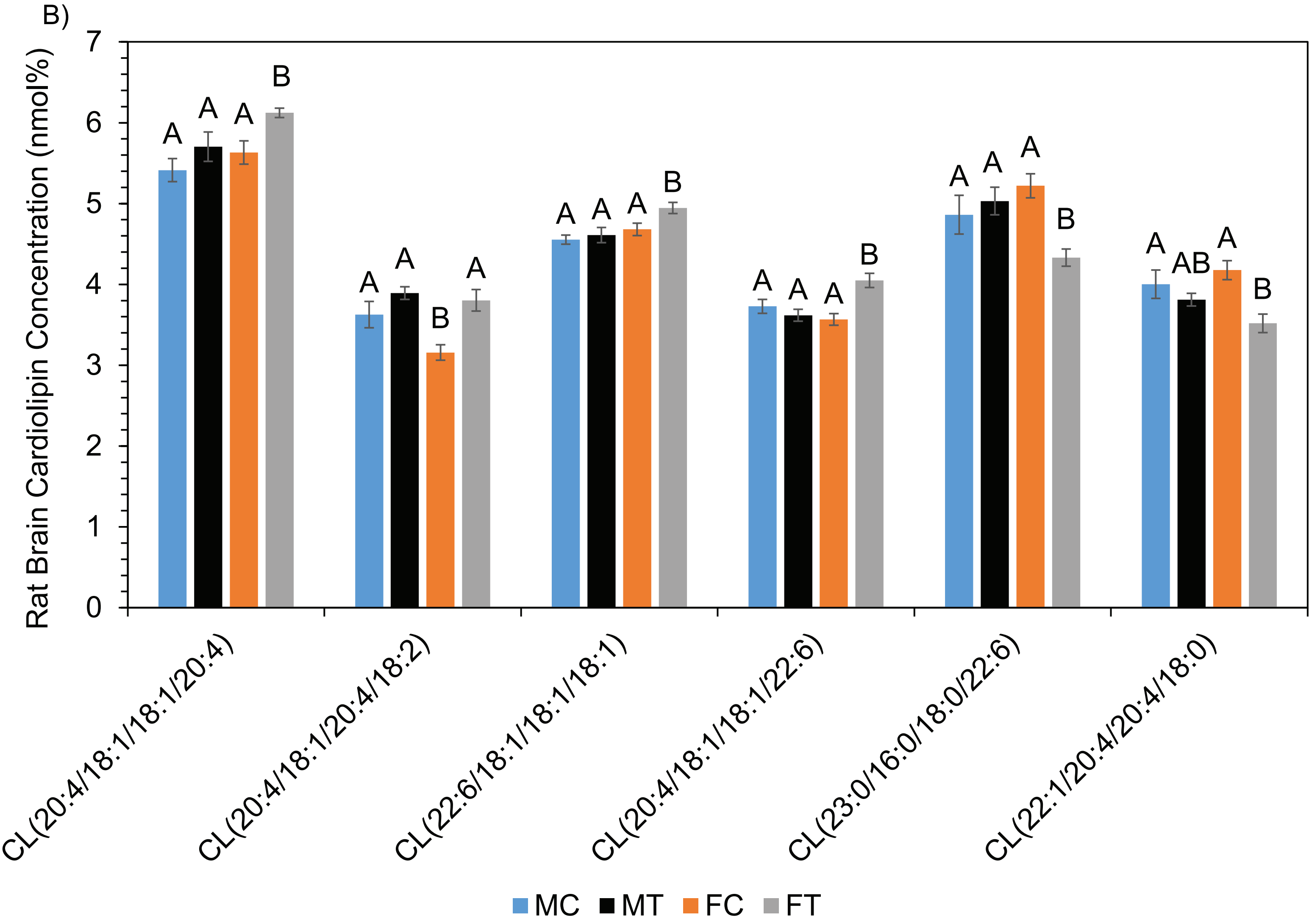

Figure S1. Alteration in cardiolipin (CL) composition after SCFA treatment in rat brain. Differential Expression (A) was used to determine significant ($p<0.05$) alterations in molecular species between treatments. Significance in two-factor control versus treated analysis indicated by two asterisks - **. This result was also graphed (B). Bar charts representitive of means ± standard error. Means represented by different superscripts are significantly different at $p<0.05$.

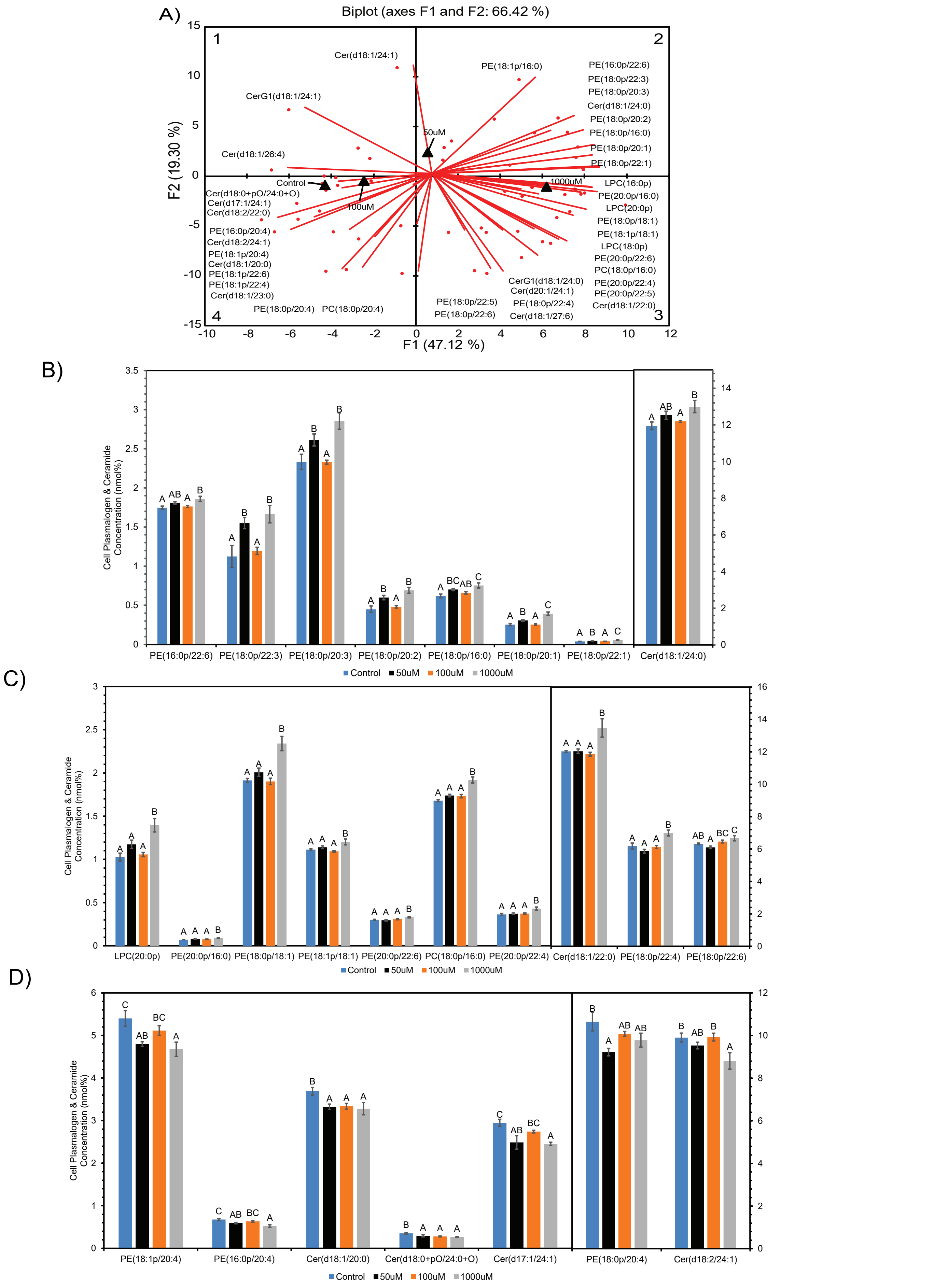

Figure S2. Alteration of plasmalogen and ceramide molecular species in neuronal cells. A) PCA biplot showing alteration in both ceramide and plasmalogen molecular species between control/100µM, 50µM, and 1000µM SCFA treatment. ANOVA was conducted based on PCA quadrants 2 (B), 3 (C), and 4 (D) corresponding to each treatment. Significant ($p<0.05$) molecular species are shown accordingly. Bar charts representitive of means ± standard error. Means represented by different superscripts are significantly different at $p<0.05$.
